# Challenges of investigating a large food-borne norovirus outbreak across all branches of a restaurant group in the United Kingdom, October 2016

**DOI:** 10.2807/1560-7917.ES.2019.24.18.1800511

**Published:** 2019-05-02

**Authors:** Mari Morgan, Vicky Watts, David Allen, Daniele Curtis, Amir Kirolos, Neil Macdonald, Ellie Maslen, Deb Morgan, Ayoub Saei, James Sedgwick, Janet Stevenson, Deborah Turbitt, Roberto Vivancos, Catriona Waugh, Chris Williams, Valerie Decraene

**Affiliations:** 1 Health Protection, Public Health Wales NHS Trust, Cardiff, United Kingdom; 2 European Programme for Intervention Epidemiology Training, Stockholm, Sweden; 3 These authors share first authorship; 4 United Kingdom Field Epidemiology Training Programme, Public Health England, London, United Kingdom; 5 Field Service - Epidemiology, National Infection Service, Public Health England, Liverpool, United Kingdom; 6 Virus Reference Department, National Infection Service, Colindale, Public Health England, London, United Kingdom; 7 National Institute for Health Research Health Protection Research Unit in Gastrointestinal Infections, United Kingdom; 8 Department of Infection Biology, Faculty of Infectious and Tropical Diseases, London School of Hygiene & Tropical Medicine, London, United Kingdom; 9 Field Service - Epidemiology, National Infection Service, Public Health England, London, United Kingdom; 10 Department of Public Health and Health Policy, NHS Lothian, Edinburgh, Scotland; 11 North East North Central London Health Protection Team, Public Health England, London, United Kingdom; 12 Incidents & Resilience Team, Food Standards Agency, London, United Kingdom; 13 Statistics, Modelling & Economics Department, National Infection Service – Data & Analytical Sciences, Public Health England, London, United Kingdom; 14 Public Health England London, Public Health England, London, United Kingdom

**Keywords:** norovirus, outbreak, restaurant chains, ready-to-eat products, United Kingdom, food-borne infections

## Abstract

During October and November 2016, over 1,000 customers and staff reported gastroenteritis after eating at all 23 branches of a restaurant group in the United Kingdom. The outbreak coincided with a new menu launch and norovirus was identified as the causative agent. We conducted four retrospective cohort studies; one among all restaurant staff and three in customers at four branches. We investigated the dishes consumed, reviewed recipes, interviewed chefs and inspected restaurants to identify common ingredients and preparation methods for implicated dishes. Investigations were complicated by three public health agencies concurrently conducting multiple analytical studies, the complex menu with many shared constituent ingredients and the high media attention. The likely source was a contaminated batch of a nationally distributed ingredient, but analytical studies were unable to implicate a single ingredient. The most likely vehicle was a new chipotle chilli product imported from outside the European Union, that was used uncooked in the implicated dishes. This outbreak exemplifies the possibility of rapid spread of infectious agents within a restaurant supply chain, following introduction of a contaminated ingredient. It underlines the importance of appropriate risk assessments and control measures being in place, particularly for new ingredients and ready-to-eat foods.

## Background

Norovirus is the predominant cause of acute gastroenteritis worldwide [1], responsible for approximately one fifth of all cases [2]. In the United Kingdom (UK) there are estimated to be 3 million sporadic episodes annually [3] and although typically mild and self-limiting [4], financial costs to patients, health services and businesses are significant [5-7].

Transmission is via the faecal-oral route [1,8], through contaminated food or water consumption or direct contact with infected persons or contaminated environments [8]. Outbreaks have been linked to contaminated shellfish [9,10], fresh produce [11-14] and ready-to-eat foods (often via infected food handlers) [15-18] frequently in restaurant settings [9,18,19].

### Outbreak detection

On 27 October 2016, Public Health England (PHE) received reports of diarrhoea and vomiting affecting 10 staff at one London branch of a restaurant group comprising 23 branches across England, Wales and Scotland (none in Northern Ireland). In the following days, customers from all branches and staff members from 22 branches reported gastrointestinal symptoms to the company head office.

Initial investigations revealed that on 26 October 2016, a new menu was introduced at all branches with over 70 dishes, 12 of which had not been served before. There were no large-scale changes in personnel or management which coincided with the outbreak.

On 1 November 2016, PHE convened an incident management team (IMT) with representation from the other UK public health agencies (Public Health Wales and Health Protection Scotland) and a joint epidemiological investigations team was established.

Here, we describe the epidemiological investigations undertaken to identify the vehicle and source of the outbreak, implement appropriate control measures and the challenges we faced in the joint investigations.

## Methods

### Epidemiological studies

#### Descriptive

The company head office compiled data on customers and staff reporting gastrointestinal illness, which they shared with environmental health departments and the IMT. Staff illness was reported by symptom onset date and customer illness by date of restaurant visit. All data, without the application of any case definitions, were used to provide a crude description of the outbreak within and across all 23 branches.

#### Analytical

Given all 23 branches were affected by gastrointestinal illness – often spread through infected food handlers and cross-contamination, it was hypothesised that a centrally distributed ingredient had become contaminated or there was a change in how the ingredient was prepared leading to contamination. To determine whether the implicated food items were the same between staff and customers of different branches, four retrospective cohort studies were conducted, one among staff of the whole restaurant group and three in customers of four branches.

The staff cohort study population included all staff employed at any UK branch between 22 and 31 October 2016. Staff tasting sessions of the 12 new menu dishes were held 24–26 October, before the public menu launch on 26 October. Staff could also eat from the main menu during their shifts. To examine the effect of exposure to food items at tasting sessions and minimise potential bias introduced through secondary transmission, in the analytical study, we included ‘early onset cases’ defined as study population members who developed vomiting or diarrhoea between 24 and 28 October and excluded ‘late onset cases’, who developed symptoms from 29 October. Individuals with a history of gastrointestinal illness since 17 October, or whose household contacts had gastrointestinal illness, were excluded.

We conducted customer cohort studies in: (i) Cardiff (branch 12), (ii) Edinburgh (branch 20) and (iii) London (branches 13 and 22), a convenience sample based on availability of epidemiological staff and customer contact details. Studies were conducted by public health agencies of the respective administrations (Public Health Wales, National Health Service (NHS) Lothian, PHE Field Service) and analysed separately. Data were combined and analysed as a single cross-administration customer cohort study, using standardised case definitions, to determine whether exposures associated with illness were common across different branches. Customer contact details were obtained from branch booking lists for the periods of interest; we did not attempt to identify customers who did not book.

Customer cases were defined as persons who ate at one of the four branches who developed severe diarrhoea (three or more episodes in 24 hours) or vomiting or two other symptoms (mild diarrhoea (less than three episodes in 24 hours), bloody stools, nausea, fever, stomach cramps and headache) within 72 hours of eating at a branch. Time frames differed by branch. Any staff members who had dined as customers were excluded. Customers with household members reporting gastrointestinal illness within 7 days before symptom onset were also excluded.

#### Questionnaire data

We developed separate but similar online questionnaires for staff and each customer study. All included questions on demographics, dining dates, symptoms, symptom onset date and the available menu items.

The staff questionnaire included questions on dishes from both the tasting and full menu. Menu items consumed in the 72 hours before symptom onset were requested for staff cases and we requested those consumed between 24 and 28 October for non-cases. The questionnaire was distributed to all staff members via email from the company management on PHE‘s behalf. Staff could complete the questionnaire between 4 and 10 November 2016 and a reminder was sent after 3 days. Staff symptoms collected during the staff cohort study were plotted by date of onset.

Customers who went to the Cardiff, Edinburgh or London (branch 22) between 26 and 28 October and to London (branch 13) between 27 and 29 October, were asked to complete the questionnaire. Timings were based on illness reports to the company and voluntary branch closure dates. Customers were contacted by telephone and email and, on consenting to participate, they were sent a link to complete the online questionnaire. They were also asked to forward it to their co-diners. Customer questionnaires were collected over ca 2 weeks and no reminder was sent. 

### Ethical statement

Ethical approval was not required as in the UK, public health agencies are able to access and use personal identifiable information for communicable disease outbreak investigations in the public interest. How the data would be utilised was outlined in the questionnaires and completion of the questionnaire was considered as implied consent.

### Data analysis

We estimated risk ratios (RR) and odds ratios (OR) as measures of association between food items consumed and being a case. We used generalised linear models to identify factors independently associated with being a case.

In the staff cohort study, exposures for tasting and full menu items were analysed separately. Food items associated with illness (RR > 1 and p < 0.1) were included in Poisson regression models with robust standard errors, constructed separately for the tasting and full menus, using a backwards stepwise approach.

For the combined customer cohort study, menu items associated with illness (RR > 1.5, eaten by at least eight cases and with 95% confidence intervals (CI) that did not include 1 were included in a logistic regression model using a backwards stepwise approach. To investigate the influence of heterogeneity between customer studies conducted at different branches, we developed an additional mixed effects logistic regression model, to estimate the association between menu items consumed and illness.

Guided by results of the multivariable analyses, we created combined variables of dishes according to common ingredients or kitchen preparation area (staff only), based upon information gathered from restaurant visits, chef interviews and national recipes provided by the company management. We repeated univariable and multivariable analysis with the combined variables.

Analysis was conducted in Stata v14.2 (StataCorp, College Station, Texas, United States) and R v.3.2.3 (R Foundation for statistical computing, Vienna, Austria).

### Other investigations

Environmental health investigations were coordinated locally for each restaurant branch by environmental health officers (EHOs), who visited branches, reviewed food hygiene procedures, took food samples and coordinated collection of faecal samples. The Food Standards Agency (FSA) conducted food chain investigations into products highlighted by the IMT including fish, shellfish, coriander, radish, chipotle and chicken products. Initial microbiological investigations of human samples were conducted in local microbiology laboratories and norovirus-positive specimens referred to the PHE Virus Reference Department (London, England) for characterisation. Food samples were tested against national standards [20] by the PHE Food, Water and Environmental Microbiology Laboratories in England.

## Results

### Epidemiological studies

#### Descriptive

**Figure 1:**
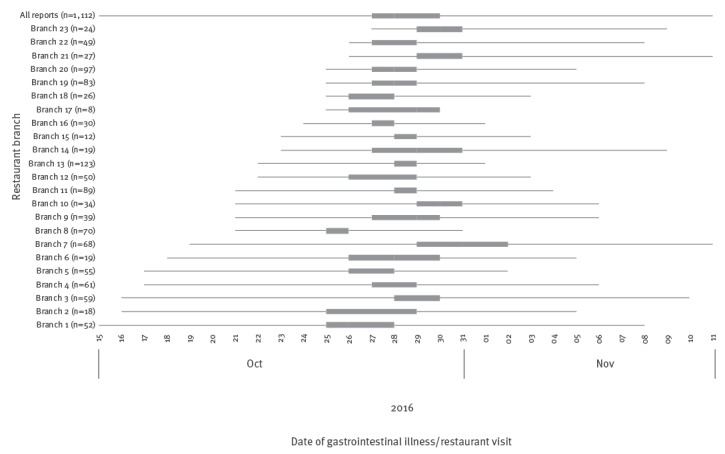
Date range of gastrointestinal illness reported by staff and customers of a restaurant group^a^, investigation of a norovirus outbreak in a restaurant group, United Kingdom, 2016

^a^ No case definitions were applied to these reports and they could include gastrointestinal illness unrelated to the norovirus outbreak and illness as a result of secondary transmission.

In total, 287 staff members and 825 customers reported gastrointestinal illness to the company. All branches had customer cases and all but one had staff cases reported. The first report of illness on 19 October was in a staff member, a growing number of staff reported illness until 25 October, when 16 staff from six branches reported illness. Staff reports peaked on 28 October, with 45 staff affected across 16 branches. The last reported illness onset date was 11 November 2016. Customers first reported illness following consumption of food at one branch on 15 October. Customer reports peaked on 29 October, with 210 customers affected across 20 branches. The last customer to report illness dined on 10 November 2016. Although there was variation between branches (Figure 1), the overall interquartile date range for staff and customer illness was 27 to 30 October.

The box reports interquartile range and whiskers report minimum to maximum date.

Gastrointestinal illness was reported to the management of the restaurant group by staff members and customers. Dates for staff were based on date of symptom onset and customer dates were based on date of restaurant visit.

### Staff cohort study

**Figure 2:**
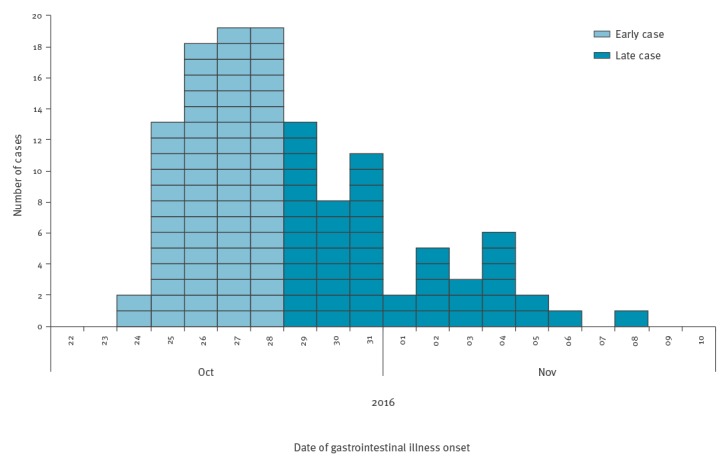
Cases of gastrointestinal illness among all restaurant staff of a restaurant group by early and late onset, staff cohort study in norovirus outbreak in a restaurant group, United Kingdom, 2016 (n = 125)

Fifty-eight percent (589/1,029) of staff completed the questionnaire, of whom a fifth (21%, 125/587) were categorised as cases. The age range of cases was 16-55 years; 57% were female. Cases were reported from 21 of 23 branches; there was large variation in attack rates between branches (0–40%). There was a sharp reduction in case numbers after 1 November 2016. The analytical study was restricted to 73 early onset cases (Figure 2), giving an attack rate of 12% (73/587). Three dishes were associated with illness in univariable analysis of each menu (Table 1) and salmon tostadas was associated with illness on the tasting menu. In multivariable analyses, illness was independently associated with consumption of salmon tostadas (RR: 2.17; 95% CI: 1.43–3.28) on the tasting menu and with chicken wings (RR: 1.75; 95% CI: 1.05–2.91) on the full menu (Table 1). Ingredient analysis showed that consuming dishes containing chipotle product A or B was independently associated with illness on analysis of each menu (RR: 2.17; 95% CI: 1.37–3.45 / RR: 1.9; 95% CI: 1.20–3.11) (Table 1); dishes containing these ingredients were consumed by 69% (50/73) and 71% (52/73) of cases, respectively. Being female was also independently associated with illness in the multivariable models for each menu and in the ingredient-based analysis (Table 1).

**Table 1:** Univariable and multivariable analysis of exposures among staff, staff cohort study in norovirus outbreak in a restaurant group, United Kingdom, 2016 (n = 73)

Exposure		Number of cases exposed	Univariable analysis			Multivariable analysis		
			RR	95% CI	p value	RR	95% CI	p value
**Winter tasting menu**								
						Model 1		
Menu item^a^	Salmon sashimi tostada	36	**2.22**	1.46–3.38	0.000	**2.17**	1.43–3.28	0.000
	Hibiscus glazed wings^b^	43	**2.01**	1.30–3.09	0.001	NA	NA	NA
	Chicken taquito^b^	33	**1.73**	1.13–2.64	0.011	NA	NA	NA
	Huitlacoche empanada^b^	37	**1.58**	1.03–2.41	0.034	NA	NA	NA
Demographics	Female	NA	NA	NA	NA	**1.78**	1.15–2.74	0.010
						Model 2		
Ingredient	Chipotle product A or B	50	**2.27**	1.42–3.60	0.000	**2.17**	1.37–3.45	0.001
Demographics	Female	NA	NA	NA	NA	**1.74**	1.12–2.68	0.013
**Full menu**								
						Model 3		
Menu item^a^	Hibiscus glazed wings	37	**2.11**	1.39–3.22	0.000	**1.75**	1.05–2.91	0.032
	Chicken taquito	28	**1.94**	1.26–2.98	0.003	**1.55**	0.92–2.63	0.101
	Huitlacoche empanada^b^	30	**1.61**	1.05–2.47	0.030	NA	NA	NA
	Salmon sashimi tostada^c^	22	**1.41**	0.89–2.24	0.141	**0.89**	0.53–1.51	0.674
Demographics	Female	NA	NA	NA	NA	**1.77**	1.14–2.74	0.011
						Model 4		
Ingredient	Chipotle product A or B	52	**2.03**	1.26–3.27	0.003	**1.90**	1.20–3.11	0.006
Demographics	Female	NA	NA	NA	NA	**1.74**	1.12–2.68	0.013

CI: confidence interval; RR: risk ratio; NA: not applicable.

^a^ Only menu items with a RR > 1.0 and p < 0.1 are displayed.

^b^ Excluded from final multivariable model.

^c^ RR > 1.0 and p > 0.1. result displayed because menu item was associated with illness on analysis of the other menu.

### Customer cohort studies

Of 159 customer responses in the combined customer cohort study (28 from Cardiff, 94 from Edinburgh and 37 from London), 37 (23%) were male and the age ranged from seven to 65 years. There were 58 (36%) confirmed cases; the attack rate ranged from 21% to 46%. Although seven menu items were associated with gastrointestinal illness in the univariable analysis (Table 2), chicken tostadas that were consumed by 57% (33/58) of cases, was the only menu item independently associated with illness in the multivariable analysis (OR: 20.65; 95% CI: 7.24–58.89). This was consistent with results of the three individual customer studies (Supplementary Table S1). Analysis of dishes containing the component ingredients of chicken tostadas identified that the ready-to-eat poached chicken (OR: 4.11; 95% CI: 1.95–8.66) and chipotle mayo (OR: 2.27; 95% CI: 1.06–4.88) were independently associated with illness. Both items were eaten by 76% (44/58) of cases. Univariable analysis of chilli components of the chipotle mayo showed both chipotle product A (RR: 2.12; 95% CI: 1.27–3.53) and chipotle product C (RR: 2.06; 95% CI: 1.24–3.44) were associated with illness; further differentiation in a multivariable model was not possible because of their frequent combined use.

**Table 2:** Univariable and multivariable analysis of menu items and ingredients eaten by customers, in combined customer cohort studies in norovirus outbreak in a restaurant group, United Kingdom, 2016 (n=58)

Exposure		No cases exposed^a^	Univariable analysis			Multivariable analysis		
			RR	95% CI	p value	OR	95% CI	p value
Menu items^b^	Chicken tostada	33	4.54	2.92–7.06	< 0.001	20.65	7.24–58.89	< 0.001
	Pork burrito	8	2.08	1.35–3.18	0.013	6.51	0.88–48.38	0.067
	Chicken taquito	10	1.82	1.16–2.85	0.029	2.55	0.59–11.11	0.212
	Pork taco	26	1.73	1.15–2.60	0.010	1.74	0.65–4.61	0.267
	Chicken taco	17	1.68	1.10–2.55	0.025	1.82	0.62–5.36	0.279
	Chorizo quesadilla^c^	13	1.58	1.01–2.47	0.066	NA	NA	NA
	Chicken quesadilla^c^	18	1.57	1.04–2.38	0.043	NA	NA	NA
Ingredients	Ready-to-eat chicken	44	2.88	1.72–4.81	< 0.001	4.11	1.95–8.66	< 0.001
	Chipotle mayo	44	2.17	1.30–3.62	0.001	2.27	1.06–4.88	0.035
	Chipotle product A^d^	44	2.12	1.27–3.53	0.002	NA	NA	NA
	Chipotle product C^d^	44	2.06	1.24–3.44	0.002	NA	NA	NA
	Chipotle product B ^e^	4	1.10	0.50–2.43	0.811	NA	NA	NA

CI: confidence interval; NA: not applicable; OR: odds ratio; RR: risk ratio.

^a^ Total number of cases = 58.

^b^ Only menu items with a RR > 1.5, CI that did not cross 1 and eaten by at least eight cases are displayed.

^c^ Excluded from final multivariable model.

^d^ Excluded because of colinearity in multivariable model.

^e^ Not significant in univariable analysis, so excluded from multivariable model.

The mixed effects logistic regression model showed that study site heterogeneity did not significantly influence the menu items associated with illness (estimated coefficient for chicken tostadas: 4.72; 95% CI: 1.81–7.63; p = 0.0015).

### Other investigations and control measures

Norovirus genogroup II.6 (GII.6) was identified from 30 of 48 samples from staff. Standard indicator organisms were not detected in any of the food samples collected.

All branches were compliant with standard hygiene regulations and EHOs reported standards to be satisfactory.

**Figure 3:**
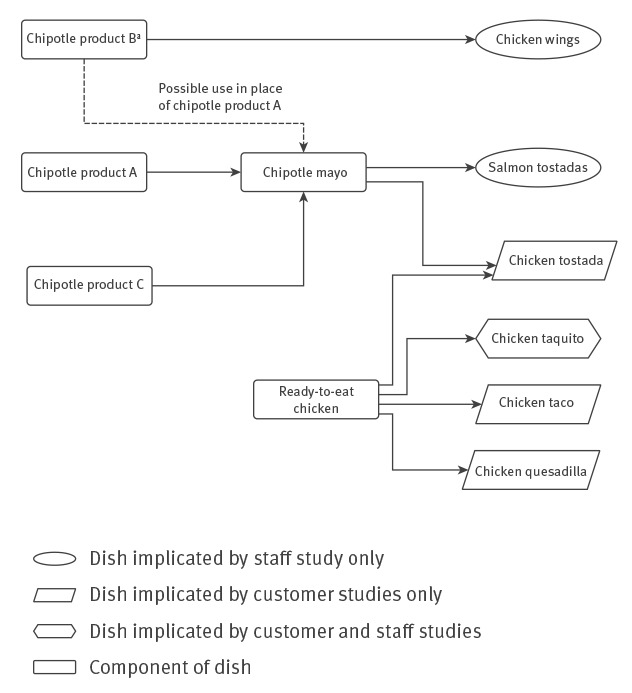
Summary of ingredient composition of menu items associated with gastrointestinal illness in customer and staff cohort studies, investigation of norovirus outbreak in a restaurant group, United Kingdom, 2016

^a ^Labelled as chipotle product A in some restaurants.

Recipes were the same for all restaurant branches. Chipotle chilli was the only ingredient in common between the two dishes implicated in the multivariable analysis of the staff cohort study and the one dish implicated by the combined customer study. Chipotle chilli was obtained from different chipotle chilli products in the three dishes. Recipes for both salmon and chicken tostadas included chipotle mayo, which contained uncooked tinned chipotle chilli product A and dried chipotle chilli product C. The recipe for the chicken wing glaze included paste chipotle chilli product B. EHOs identified that in some branches product B had been labelled as product A (Figure 3).

Trace-back investigations identified that chipotle product B was newly imported from outside the European Union. Cook-chill and food safety management for the two chicken suppliers were satisfactory and the product was also distributed to other restaurants in the UK.

A central kitchen in London supplied some components of dishes for London restaurants only. Restaurant service kitchens in all branches were divided into three sections; all dishes implicated by multivariable analyses (staff and customer studies) were prepared in the salad section.

The company voluntarily closed 16 branches in the UK (the first on 26 October) and discarded fresh and partially used produce. In addition, the branches carried out deep cleaning of their restaurants to remove any norovirus contamination, including environmental fogging. Staff were advised to stay off work for 72 hours following their last gastrointestinal symptom and were offered paid sickness absence to encourage policy adherence. All potential vehicles of norovirus transmission identified by the epidemiological studies were removed from the menu.

## Discussion

We describe the largest norovirus restaurant outbreak recorded in the UK to date. Coincident gastrointestinal illness in multiple branches suggested a point source outbreak, although later in the outbreak staff cases were likely due to secondary transmission. Concurrence with a national menu change and the involvement of all 23 branches in the restaurant group, suggested the vehicle of transmission was a nationally distributed item that had either been newly introduced to the menu or had been modified for use in the new menu. The sharp reduction in cases suggested the vehicle had either been withdrawn, destroyed or used. There were no similar reports of illness associated with other UK food outlets, suggesting the contaminated product batch was unique to this restaurant group.

Three separate chipotle products were ingredients of dishes associated with gastrointestinal illness. Chipotle product A was a tinned constituent ingredient of the chipotle mayo, independently associated with illness in the combined customer study and served uncooked with both salmon and chicken tostadas. However, it was not a new ingredient and it was biologically implausible as the vehicle since norovirus would be inactivated by the canning process [21]. Environmental health investigations identified a similar product known as chipotle product B, which had been labelled as chipotle product A in some branches; this was a constituent ingredient of the chicken wings implicated in the staff cohort study. It had been newly imported from outside the European Union for the new menu, was not tinned and was not cooked during initial processing. It is plausible, therefore, that chipotle product B may have been mistakenly used in the chipotle mayo in place of chipotle product A in some branches and this may explain the variation in attack rate. Although implicated in customer cohort studies, chipotle product C was not considered a likely vehicle as it was boiled for 15 minutes before use, which would have destroyed norovirus.

Customer cohort studies observed a stronger association between gastrointestinal illness and consumption of dishes containing poached, ready-to-eat, vacuum-packed chicken from a new supplier, which had been introduced for the menu change. This product was only used once the chicken from the previous supplier had run out – possibly explaining the staggered symptom onsets in branches. This chicken product was also used in other dishes, but was only served without further reheating in the chicken tostadas. The food chain investigations found no evidence to implicate this chicken product as a vehicle for norovirus transmission. In addition, it was supplied to other UK restaurants, but there were no reports of similar outbreaks or reports of gastrointestinal illness from staff responsible for processing the chicken (data not shown).

There are plausible routes of contamination for chipotle product B and the ready-to-eat chicken, before they were received and distributed around the restaurant group. Fresh produce has been implicated in multiple norovirus outbreaks [11-14]. Food items can become contaminated during cultivation, harvesting or processing, usually as a result of contact with contaminated sewage or infected food handlers. Ready-to-eat meat products have also been implicated as vehicles for norovirus outbreaks [22] contaminated directly by food handlers during processing [23]; the capacity for contamination via slicing equipment has also been demonstrated [22]. In this investigation, we were unable to test any of the potential vehicles identified for norovirus, as accredited tests are only available for limited food items, not implicated in this outbreak. Development of sensitive laboratory methods for testing food specimens for viruses during outbreaks would be valuable in future investigations.

Introduction of contaminated fresh produce [24-27] or ready-to-eat foods [28] from a single supplier has been implicated in several food-borne outbreaks. All dishes associated with gastrointestinal illness in the multivariable analysis were prepared in the salad section, meaning that cross-contamination there could have played a role in transmission. This finding may explain the variation found between the customer and staff studies, as well as between the customer cohort studies conducted in different branches. This finding may also partially explain why staff cases were almost twice as likely as non-cases to be female; studies have shown that women are more likely to choose salad items than men [29].

This outbreak and others affecting restaurant chains [24,30,31] highlight the speed with which pathogens can spread over wide geographical areas, when one or more contaminated ingredients enter a national restaurant supply chain and appropriate risk assessment and controls are not in place. The public health implications could have been much more serious had this outbreak been caused by a more virulent pathogen than norovirus, which is generally a mild and self-limiting illness. Both food products implicated by the epidemiological investigations were highlighted to the restaurant group management and they have since reported working with suppliers to minimise the risk of further outbreaks. 

### Challenges

There were many challenging aspects to the epidemiological investigations. The descriptive data for the entire outbreak was collected by the company and had no case definitions applied. This means that secondary cases and gastrointestinal illness unrelated to the outbreak may have been included as cases. Reporting of gastrointestinal illness may also have been influenced by high media coverage, potentially explaining reports of illness before the introduction of the new menu. The menu included a large number of dishes and was complex; dishes had many ingredients and garnishes and ingredients were often common to multiple menu items. In addition, the menu was designed for customers to share dishes, many with similar names, which could have made it more difficult for customers to distinguish and accurately recall what they had eaten. The high media interest and publicity regarding the outbreak may have encouraged customers to exaggerate symptoms, potentially misclassifying non-cases as cases. Introducing paid sickness absence may similarly have inflated staff case numbers.

The incident management team was supplied with standardised recipe cards that detailed the ingredients used, preparation instructions and photos of the dishes, which supported a recipe-based cohort study design (used in the fenugreek sprout *Escherichia coli* O104:H4 outbreak in Germany [32]). However, within the 23 branches, there may have been local undocumented variation in how a dish was prepared and different products were also observed with the same name; both of these could have introduced inaccuracies into our ingredient analyses. Finally, although coordinated centrally, customer studies were carried out by three different agencies, each with small sample sizes representing only a proportion of customers exposed [33]. While we endeavoured to standardise methodology between the agencies, there were some local differences in practice.

### Conclusions

This outbreak demonstrates that an entire restaurant group can be affected within a short time frame by the introduction of a contaminated ingredient. The investigation highlights the challenges in identifying the vehicle of transmission from a large, complex menu with multiple ingredients used in numerous dishes with possible undocumented variation between branches. In hindsight, a more coordinated approach to conducting the epidemiological studies may have achieved a more coherent outcome. For example, a single study incorporating both staff and customers from across the restaurant group would have been more logistically challenging to set up, but results may have been easier to interpret. This is a learning point for cross-UK outbreak management for the future.

We recommend that multi-branch restaurants with central suppliers and kitchens are vigilant to the possibility of contaminated ingredients entering their supply chain and the potential for rapid spread of pathogens. Food business operators should ensure that appropriate hazard analysis and critical control point processes are in place, particularly for new ingredients and ready-to-eat foods and consider the potential for cross-contamination within preparation areas in risk assessments.

## Supplementary Data

238000 Supplementary Table S1
