# Supplementary material for: Challenges of investigating a large food-borne norovirus outbreak across all branches of a restaurant group in the United Kingdom, October 2016
Source: Euro Surveill. 2019 May 2;24(18):1800511. doi: 10.2807/1560-7917.ES.2019.24.18.1800511 (PMC6505182; doi:10.2807/1560-7917.ES.2019.24.18.1800511)
Supplement: Supplementary Table S1 [file 1800511_MORGAN_SupplementaryTableS1.pdf]

Univariable and multivariable analysis of menu items and ingredients eaten by customers in the individual customer studies[a], investigating an outbreak of norovirus in a restaurant group in the United Kingdom, 2016

This supplementary material is hosted by Eurosurveillance as supporting information alongside the article 'Challenges of investigating a large foodborne norovirus outbreak across all branches of a restaurant group in the UK, October 2016' on behalf of Morgan M., Watts V. et al who remain responsible for the accuracy and appropriateness of the content. The same standards for ethics, copyright, attributions and permissions as for the article apply. Eurosurveillance is not responsible for the maintenance of any links or email addresses provided therein.

				Univariable analysis			Multivariable analysis		
**Study**	**Exposure**		**% Cases exposed[b]**	**Risk/ Odds Ratio**	**95% CI**	**p-value**	**Odds Ratio**	**95% CI**	**p-value**
**Branch 12 case control study[b]**	**Menu items** (OR> 1 & eaten by at least 3 cases)	Chicken tostada	53.8	28.0	2.5 – 1310	<0.0001		Not done	
		Chicken taco	38.5	15.0	1.3 – 738	0.006			
		Chorizo frijoles	23.1	7.20	0.5 – 393	0.069			
		Prawn tacos	38.5	4.58	0.7 – 35.0	0.058			
		Chorizo quesadilla	23.1	3.45	0.3 – 45.7	0.192			
		Churros y chocolate	53.8	2.48	0.5 – 12.2	0.191			
		Chicken taquito	23.1	.	1.7 – .	0.012			
	**Ingredients[b]**	Ready-to-eat chicken	84.6	9.78	1.5 – 103.28	0.004		Not done	
		House dressing	53.8	3.69	0.71 – 19.34	0.066			
		Chipotle mayo	53.8	3.00	0.60 – 15.13	0.117			
		Chipotle product C	53.8	3.00	0.60 – 15.13	0.117			
		Chipotle product A	69.2	2.08	0.42 – 11.57	0.307			
		Chipotle product B	7.7	0.96	0.02 – 20.23	0.973			
**Branch 20 cohort study**	**Menu items** (RR>1.5 & eaten by at least 4 cases)	Chicken tostada	58.5	2.92	1.87 – 4.54	<0.001		Not done	
		Pork burrito	12.2	2.42	1.88 – 3.10	0.036			
		Chicken taquito	14.6	2.08	1.40 – 3.09	0.06			

		Tortillas	68.3	1.97	1.18 – 3.31	0.005			
		Grilled chicken	9.8	1.88	1.14 – 3.11	0.239			
		Guacamole	48.8	1.70	1.10 – 2.64	0.036			
		Chicken quesadilla	31.7	1.67	1.08 – 2.58	0.068			
	**Ingredients**[c]	Ready-to-eat chicken	78.0	2.99	1.61 – 5.53	<0.001		Not done	
		Chipotle	87.8	1.63	0.75 – 3.54	0.262			
		Chipotle mayo	78.0	1.56	0.86 – 2.81	0.175			
		Chipotle product C	78.0	1.48	0.82 – 2.66	0.243			
		Radish	78.0	1.37	0.74 – 2.37	0.439			
		Chipotle product A	85.4	1.05	0.55 – 2.01	1			
		Avocado	85.4	1.05	0.55 – 2.01	1			
		Chipotle product B	19.5	0.87	0.48 – 1.58	0.834			
**Branch 13 and 22 cohort study**	**Menu items** (OR>1& p value ≤0.2)	Chicken tostada	36.4	16.59	1.88 - ∞	0.005	5.07	0.53 - ∞	0.0024
		Rice & beans (side)	18.2	6.16	0.46 - ∞	0.083	6.16	0.25 - ∞	0.014
	**Ingredients**	Not done						Not done	

a. Similar methods for data collection and analysis were used in each but there were some variations in definitions, in dishes included in combined variables (based on local restaurant intelligence) and analysis packages used.

b. Initially conducted as a cohort study with participants recruited from booking lists only. Because of low numbers, study design amended to case control with additional recruitment of participants from lists held by environmental health department. For the combined customer cohort analysis, only those recruited from the booking lists were included.

c. Menu items including the cooked chicken product were identical between Cardiff and Edinburgh, but some differences between menu items included in other combined variables. For combined customer cohort study, composite variables were created based on national recipe cards.
